# Exploration of the causal relationship between inflammatory cytokines and prostate carcinoma: a comprehensive Mendelian randomization study

**DOI:** 10.3389/fonc.2024.1381803

**Published:** 2024-08-29

**Authors:** Xianfu Cai, Decai Wang, Chenguang Ding, Yang Li, Jin Zheng, Wujun Xue

**Affiliations:** ^1^ Department of Renal Transplantation, the First Affiliated Hospital of Xi’an Jiaotong University, Xi’an, China; ^2^ Department of Urology, Mianyang Hospital Affiliated to School of Medicine, University of Electronic Science and Technology of China Mianyang Central Hospital, Mianyang, China

**Keywords:** inflammatory factors, prostate carcinoma, risk, malignant neoplasm of prostate, Mendelian randomization

## Abstract

**Background:**

Prostate cancer (PCa) is one of the most prevalent malignancies affecting males; however, the role of inflammatory activity in the pathogenesis of this disease is not yet fully elucidated. Although inflammation is recognized as being closely associated with the onset and progression of PCa, the specific causal relationships between individual inflammatory factors and the disease require further clarification.

**Methods:**

Mendelian randomization (MR) methodologies can mitigate bias by utilizing whole-genome sequencing data, leveraging specific genetic variants to assess causal relationships between a given exposure and an outcome of interest. This research employed an MR approach to investigate the association between inflammatory cytokines and PCa.

**Results:**

In total, 44 inflammatory cytokines were evaluated in a large GWAS dataset to enable the drawing of robust conclusions. Elevated circulating C-reactive protein (CRP) and prostaglandin E2 (PGE-2) levels were related to greater PCa risk. The reverse Mendelian randomization (MR) study indicates a causal relationship between prostate cancer and stem cell factor (SCF) (*P*=0.025).

**Conclusion:**

CRP and PGE-2 play crucial roles in the regulation of PCa development. Moreover, PCa may have an impact on SCF levels. Further research is imperative to elucidate whether these biomarkers can be effectively utilized to prevent or treat PCa.

## 1 Introduction

Following non-melanoma skin cancer, prostate cancer emerges as the most prevalent condition. It ranks second in contributing to cancer-related mortality among males in the United States. In 2022 alone, there were 268,500 diagnoses and 34,500 deaths from PCa (1). PCa incidence and progression are shaped by various factors. Recently, multiple studies have demonstrated the crucial role that inflammatory activity plays in PCa onset and progression (2), with both acute and chronic forms of inflammation playing relevant contributing roles in this context (3).

Sustained increases in inflammatory activity and associated genetic changes can contribute to damage to cells, leading to malignant disease progression. Cytokines play a role in all inflammatory processes and the onset and development of cancer (4). Ongoing prostate tissue damage can be driven by greater infiltration by B cells, T cells, and tumor-associated macrophages, establishing a pro-inflammatory cytokine- and growth factor-rich tumor microenvironment (5–7). While the release of inflammatory mediators is closely related to tumorigenesis and cancer progression (8), inflammation is a complex and multifaceted entity that is not always conducive to tumor development. Indeed, inflammation can play a dual role by suppressing or facilitating tumor advancement depending on cellular composition and the particular nature of the associated immune response (9). There is ample evidence that variants in genes encoding factors associated with inflammatory activity, such as IL-4, IL-6, COX-1, COX-2, CCL-2, and CCL-5, can modulate the production of the related proteins, thereby influencing the risk of PCa in carriers of these variants (10). The precise nature of the role of inflammatory factors in PCa thus remains controversial given the lack of clarity regarding the nature of this relationship, and there is a need for additional research to clarify whether they are causally linked. Studies focused on the precise interplay between particular inflammatory mediators and PCa are thus of clear clinical value.

MR studies use genetic variants as instrumental variables to explore causal links between risk factors and health outcomes. Unlike traditional observational methods, MR studies are more robust against confounding, measurement errors, and reverse causation. This robustness stems from the inherent random assignment of genetic alleles at conception, mitigating potential biases in causal inference. As such, these analyses can provide more accurate evidence suitable for causal inferences (11). A two-sample MR analysis was undertaken using genetic variants as IVs to determine the causal association of inflammatory cytokine levels with the risk of PCa. As there is no causal association between potential confounders and genetically predicted inflammatory mediator levels, exposures were expected to fully mediate the relationship between the selected genetic variants and PCa. To probe these relationships, relevant variants were initially extracted from the summary data of genome-wide association studies (GWAS) about the levels of 44 inflammatory cytokines, of which 41 had been reported previously (12) as well as 3 that have been advanced as factors potentially related to inflammatory activity in the literature. Subsequently, we conducted a Mendelian randomization (MR) study to examine the causal association between 44 inflammatory biomarkers and prostate cancer.

## 2 Methods

### 2.1 Instrumental variable selection

Genetic analyses were undertaken for 44 inflammatory mediators, including prostaglandin E2, procalcitonin, C-reactive protein, and 41 other inflammatory markers.

**Figure 1:**
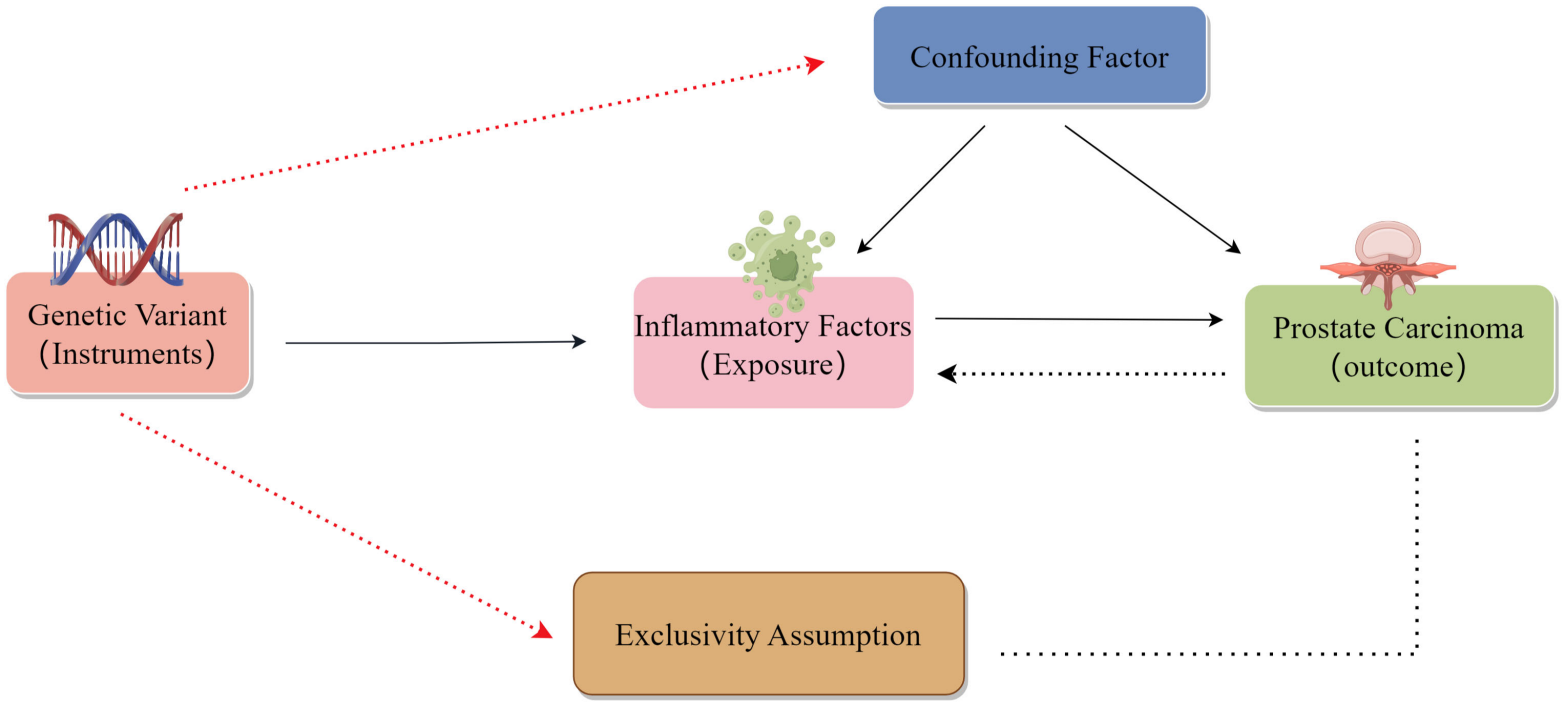
Schematic overview of the present two-sample MR analysis approach.

To be successful, MR analyses rely on three major assumptions: relevance, independence, and exclusion restrictions (13). Relevance is based on the assumption that the variants chosen as genetic instruments are related to risk factors of interest, with these variants also being assumed to be independent of any confounding factors linking the risk factors to the outcome of interest. The exclusion restriction assumption is that the variants are only related to the outcome via the indicated risk factor and not through any other pathways. For the present two-sample analysis, two GWAS datasets were used to select genetic variants significantly related to 44 inflammatory cytokines and PCa (**Figure 1**). Initially, SPs strongly associated with inflammatory cytokines and PCa were identified at a genome-wide significance threshold of *P* < 5x10^-8^. Still, a less restrictive cut-off (*P* < 5x10^-6^) was selected as some cytokines exhibited few SNPs at the initial cut-off. SNPs were clumped together to minimize the risk of linkage disequilibrium (10,000 kb, r^2^ = 0.001). The exclusion of palindromic SNPs was warranted due to the challenge of indicating their alignment in a consistent direction for both the exposure and outcome in the GWAS datasets. Then, R^2^ values for individual SNPs were used to calculate the variance proportion of exposure. F-statistic values were estimated to avoid bias from weak genetic instruments (14, 15). For any SNPs that were not available in summary results, proxy SNPs (R^2^ > 0.9) from LDlink (https://ldlink.nci.nih.gov/) were instead used (16).

### 2.2 Data sources

In the context of this two-sample MR investigation, the initial phase involved curating pertinent genetic variants from publicly accessible GWAS databases, precisely selected to function as IVs for the study’s diverse exposures and outcomes. SNPs associated with inflammatory cytokines were extracted from a comprehensive study involving 8,293 cases, systematically evaluating 41 distinct circulating cytokines (12), applying C-reactive protein, procalcitonin, and prostaglandin E2. The summary statistics concerning malignant neoplasms of the prostate were meticulously derived from the UK Biobank, featuring a cohort comprising 3,436 cases and 459,574 control subjects of European ancestry. A comprehensive enumeration of data sources is elaborated in **Table 1**.

**Table 1:** Data sources.

Data Source	Database	Data type	Detailed information
**Inflammatory cytokines**	European Bioinformatics Institute	RNA-seq data	RNA-seq data with a total of 376,023 samples and 472,850,322 SNPSs.
**Malignant neoplasms of the prostate**	UK Biobank	RNA-seq data	The total sample size is 463,010, including 3,436 cases and 459,574 controls.

SNPs and their respective summary data were limited exclusively to individuals of European ancestry (17) to mitigate the potential influences of population stratification bias on the outcomes. Details regarding the included cytokines based on GWAS summary data are presented in **Table 2**.

**Table 2:** Sample sizes for analyzed cytokines derived from GWAS studies.

Cytokines	Abbreviation	Sample size	Number of SNPs	GWAS ID	Year
Monocyte chemotactic protein-1 (CCL2)	MCP1	8,293	9,801,908	ebi-a-GCST004438	2016
Stem cell growth factor beta	SCGFβ	3,682	9,574,890	ebi-a-GCST004428	2016
Vascular endothelial growth factor	VEGF	7,118	9,784,803	ebi-a-GCST004422	2016
Platelet-derived growth factor-BB	PDGFbb	8,293	9,800,009	ebi-a-GCST004432	2016
Eotaxin (CCL11)	Eotaxin	8,153	9,793,404	ebi-a-GCST004460	2016
Macrophage migration inhibitory factor (glycosylation-inhibiting factor)	MIF	3,494	9,537,573	ebi-a-GCST004423	2016
TNF-related apoptosis-inducing ligand	TRAIL	8,186	9,698,525	ebi-a-GCST004424	2016
Hepatocyte growth factor	HGF	21,758	12,703,547	ebi-a-GCST90012045	2020
Growth regulated oncogene-α (CXCL1)	GROα	3,505	9,528,505	ebi-a-GCST004457	2016
Interferon gamma-induced protein 10 (CXCL10)	IP10	3,685	9,576,881	ebi-a-GCST004440	2016
Tumor necrosis factor-beta	TNFβ	1,559	6,304,298	ebi-a-GCST004425	2016
Tumor necrosis factor-alpha	TNFα	3,454	9,500,449	ebi-a-GCST004426	2016
Stromal cell-derived factor-1 alpha (CXCL12)	SDF1α	5,998	9,736,366	ebi-a-GCST004427	2016
Stem cell factor	SCF	21,758	13,102,995	ebi-a-GCST90012023	2020
Regulated on activation, normal T cells expressed and secreted (CCL5)	RANTES	3,421	9,523,827	ebi-a-GCST004431	2016
Macrophage inflammatory protein-1β (CCL4)	MIP1β	8,243	9,802,973	ebi-a-GCST004433	2016
Macrophage inflammatory protein-1α (CCL3)	MIP1α	3,552	9,519,267	ebi-a-GCST004434	2016
Monokine induced by interferon-gamma (CXCL9)	MIG	3,685	9,579,894	ebi-a-GCST004435	2016
Macrophage colony-stimulating factor	MCSF	840	9,184,521	ebi-a-GCST004436	2016
Interferon-gamma IFN-γ	IFN-γ	7,701	9,785,363	ebi-a-GCST004456	2016
Basic fibroblast growth factor	bFGF	7,565	9,790,946	ebi-a-GCST004459	2016
Granulocyte colony-stimulating factor	GCSF	7,904	9,788,961	ebi-a-GCST004458	2016
C-reactive protein levels	CRP	9,544	23,258,877	ebi-a-GCST005067	2017
Procalcitonin	PCT	3,301	10,534,735	Prot-a-344	2018
Prostaglandin E synthase 2	PGE-2-2	10,708	15,566,910	ebi-a-GCST90019440	2016
Interleukin-1 receptor antagonist	IL1ra	21,758	13,081,270	ebi-a-GCST90012004	2020
Interleukin-1-beta	IL-1β	3,309	9,983,642	ebi-a-GCST004448	2016
Interleukin-2	IL-2	3,475	9,512,914	ebi-a-GCST004455	2016
Interleukin-4	IL-4	8,124	9,786,064	ebi-a-GCST004453	2016
Interleukin-5	IL-5	3,364	9,450,731	ebi-a-GCST004452	2016
Interleukin-6	IL-6	21,758	11,782,139	ebi-a-GCST90012005	2020
Interleukin-7	IL-7	3,409	9,692,306	ebi-a-GCST004451	2016
Interleukin-8	IL-8	21,758	12,717,989	ebi-a-GCST90011994	2020
Interleukin-9	IL-9	3,634	9,567,876	ebi-a-GCST004450	2016
Interleukin-10	IL-10	7,681	9,793,445	ebi-a-GCST004444	2016
Interleukin-13	IL-13	3,557	9,539,073	ebi-a-GCST004443	2016
Interleukin-16	IL-16	3,483	9,551,485	ebi-a-GCST004430	2016
Interleukin-17	IL-17	7,760	9,786,653	ebi-a-GCST004442	2016
Interleukin-18	IL-18	21,758	13,102,515	ebi-a-GCST90012024	2020
Interleukin-12p70	IL-12p70	8,270	9,799,886	ebi-a-GCST004439	2016
Interleukin-2 receptor, subunit alpha	IL2rα	10,708	15,567,202	ebi-a-GCST90019431	2020
Beta nerve growth factor	βNGF	21,758	11,675,444	ebi-a-GCST90012042	2020
C-X-C motif chemokine 16	CXCL16	21,758	13,144,526	ebi-a-GCST90012059	2020
C-C motif chemokine 27	CTACK	3,301	10,534,735	Prot-a-405	2018

The sample size of the analyzed cytokines in this study was derived from GWAS.

### 2.3 MR analyses

The examination of potential causal relationships was undertaken employing the inverse variance-weighted (IVW) method. While this approach can be efficiently implemented with high statistical power, it erroneously assumes that all genetic variants are valid IVs even when this is not the case (18). Alternative approaches with enhanced robustness, exempt from the need for such assumptions, were consequently employed to yield more consistent estimations of causal relationships. The weighted median approach can produce effective estimates provided more than half of the weights are associated with valid IVs, thus endowing this approach with greater tolerance for invalid IVs (19). The MR-Egger method employs the instrument strength independent of the direct effect (InSIDE) assumption to provide an alternative means of consistently estimating causal effects. The MR-Egger regression intercept was assessed to test for potential directional pleiotropy, with P < 0.05 being considered significant (20).

When the heterogeneity-related I^2^ statistic that measures the violation of the non-overlapping measurement error (NOME) assumption for IVs is low (I^2^ < 90%), significant regression dilution may still arise. When this NOME assumption is violated, the SIMEX method can be employed to reduce the associated bias (19). The ‘mr_egger’ function within the ‘TwoSampleMR’ package was used to conduct MR-Egger regression, and the ‘mr_raps’ function was used for SIMEX adjustments. Simple mode and weighted model analyses were also conducted as sensitivity analyses. The MR-PRESSO method can identify abnormal genetic variants exhibiting horizontal pleiotropy provided > 50% of the instruments utilized are valid (21). The F-statistic is an approximation based on summary-level data that assesses the relevance of exposure-related IVs. An F-statistic > 10 is regarded as sufficiently strong to minimize the risk of bias from weak IVs. Heterogeneity among SNP estimates for IVW analyses was estimated with ‘Cochran’s Q test.

When MR-PRESSO analyses yield any abnormal SNPs, these are excluded, while the remaining IVs are assessed to establish the most appropriate analytical strategy. The ‘mr_presso’ function within the MR-PRESSO package was used for this analysis. After selecting an approach, the remaining analytical approaches are used to conduct sensitivity analyses when probing potential causal relationships.

When the SNPs utilized as IVs exhibited missing exposure- or outcome-related summary data, they were omitted from these analyses. The R TwoSample MR package (version 4.3.2) (22) and the MR-PRESS tool (21) were used to conduct these analyses. Pre-registration for this study was not undertaken on any platform.

## 3 Results

At a genome-wide significance threshold of 5x10^-8^, just 8 of the 44 selected inflammatory factors exhibited three or more genetic variants that were valid instruments. In contrast, a less stringent threshold (*P* < 5x10^-6^) was employed for the remaining cytokines to ensure sufficient SNPs to permit additional MR analysis. These SNPs were associated with proportions of explained variance for the corresponding inflammatory cytokines ranging from 0.1-15%, and all exhibited F-statistic values greater than 10 such that they were unlikely to be impacted by weak instrument bias (**Supplementary Table S1**).

The primary analysis of the relationship between these 44 inflammatory factors and PCa was conducted using an IVW method in all cases, given the absence of evidence of weak IVs or heterogeneity (23). No heterogeneity was detected for IL-12p70 or IFN-γ, while the same was not true for PGE-2 as it could not be assessed owing to the limited number of SNPs. However, the heterogeneity test for PGE-2 did not exhibit any significant heterogeneity (*P* = 0.67).

**Figure 2:**
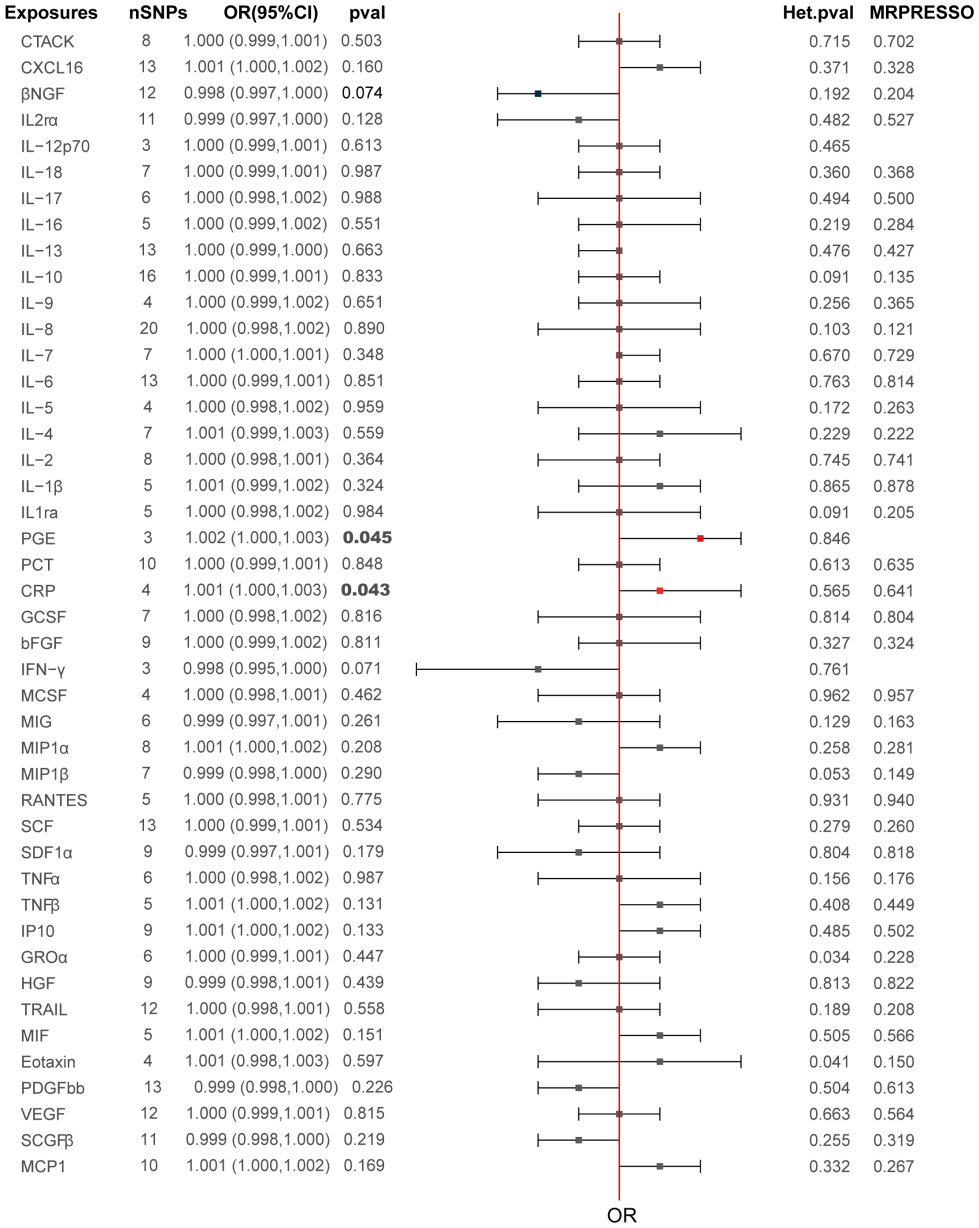
Causal relationships between the 44 inflammatory factors and PCa.

**Figure 3:**
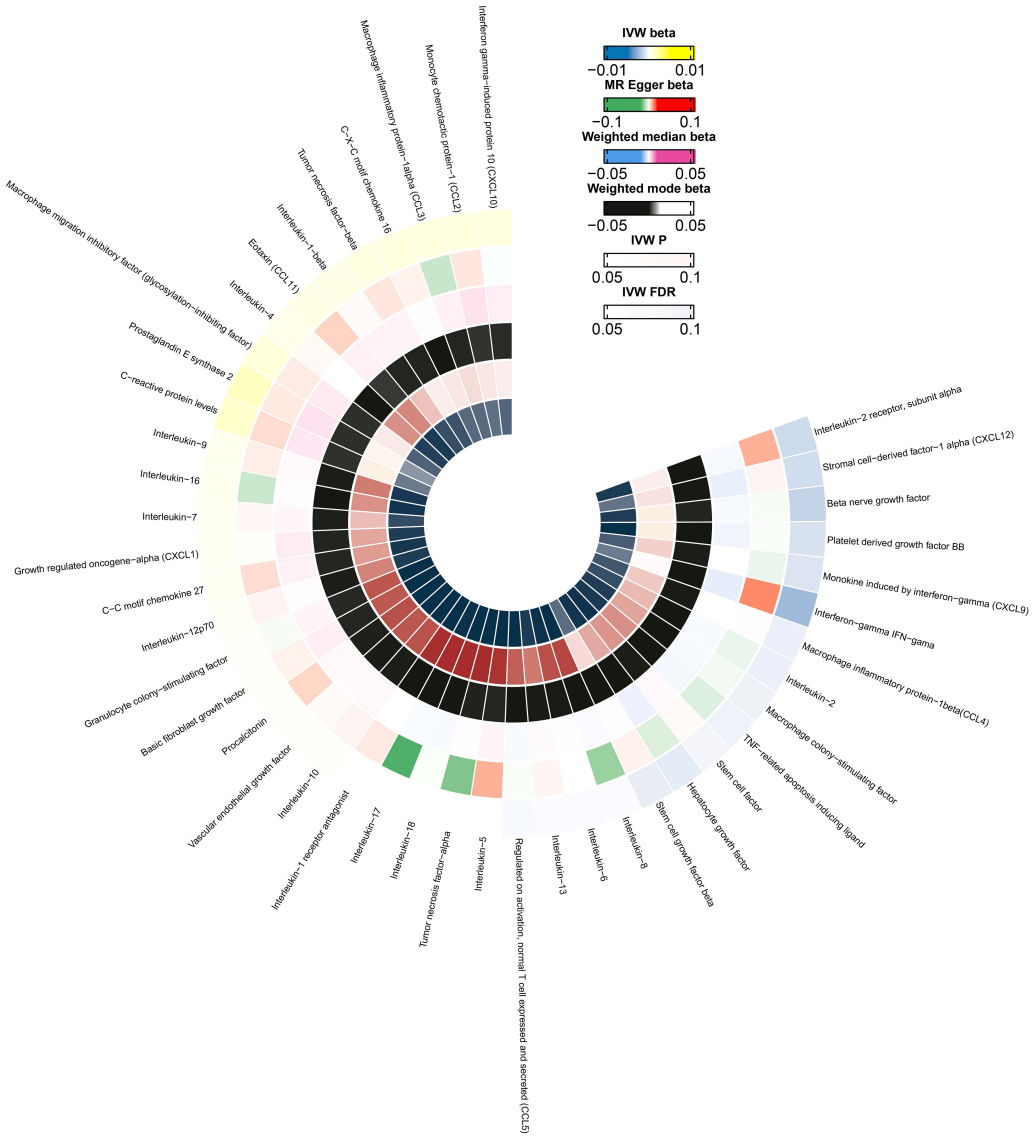
A circle diagram representing the odds associated with different MR analytical methods for the 44 evaluated cytokines. The innermost and outermost circles represent FDR and IVW, respectively. Different probabilities are denoted with different colors.

**Figure 4:**
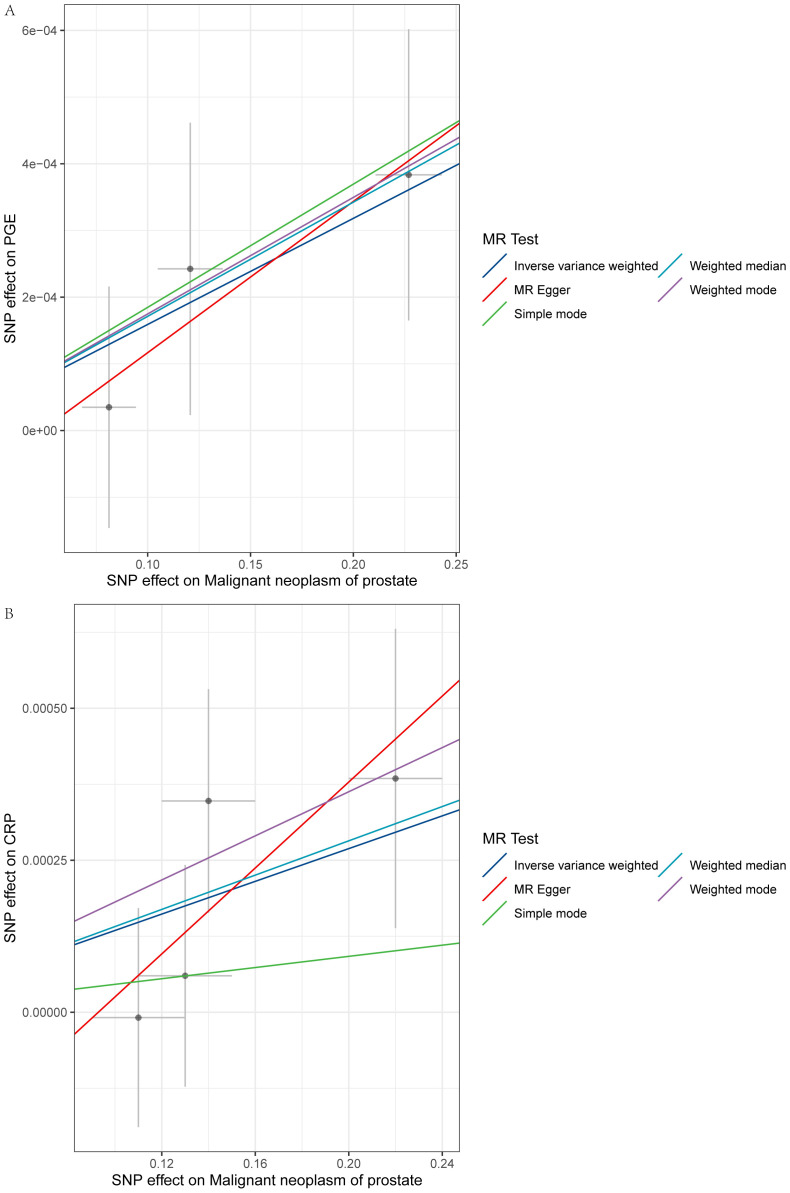
Scatter plots for the MR analyses of the relationship between PCa and inflammatory cytokines. Individual inverse variance associations with PCa risk and cytokines are represented with black dots. The vertical and horizontal lines correspond to the odds ratios and 95% confidence intervals for each IV. The causal effect estimated with the MR method is represented by the slope of the line (**A**: PGE-2, **B**: CRP).

**Figure 5:**
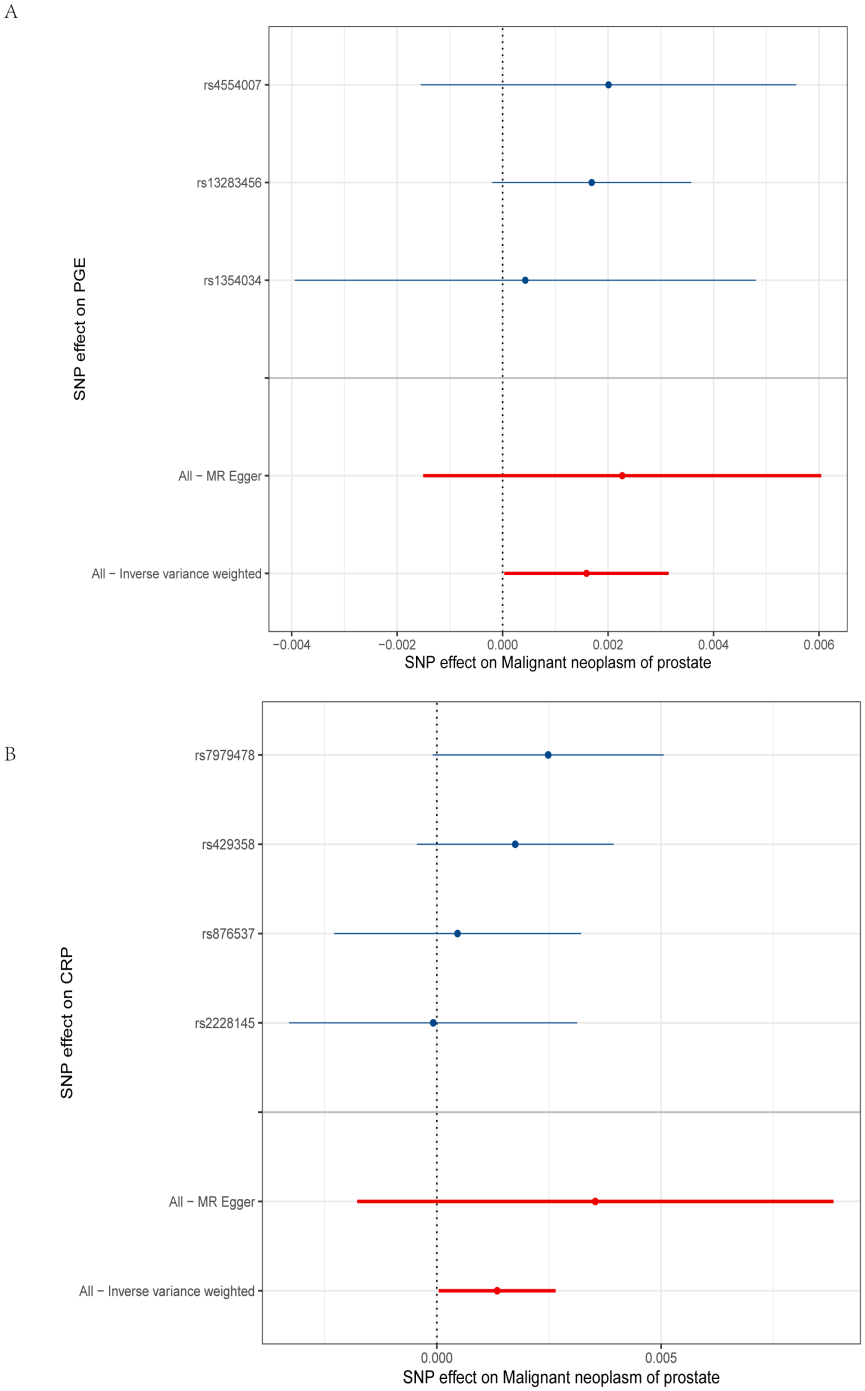
Forest plot of PGE-2 and CRP. **(A)** The y-axis represents SNPs strongly related to PGE-2, while the x-axis represents the increased risk of malignant prostate tumors; **(B)** The y-axis represents SNPs strongly related to CRP, while the x-axis represents the increased risk of malignant prostate tumors.

The primary MR analysis’s main results are in **Figures 2**, **3** and **Supplementary Table S2**. A positive association was detected between genetically determined high CRP levels (increased by one SD) and a 0.1% increase in the odds of PCa (OR: 1.001, 95% CI: 1.000-1.003, *P* = 0.043) as determined using the IVW method. A similar, albeit non-significant, trend was also observed using the weighted median method (OR: 1.001, 95% CI: 1.001-1.003, *P* = 0.072). MR-Egger analyses failed to detect any significant relationship, although a similar general trend was noted (OR: 1.003, 95% CI: 1.000-1.008, *P*= 0.32). Significant associations between inflammatory cytokines and prostate cancer, specifically involving CRP and PGE, are detailed in **Table 3**. A causal association between PGE-2 and PCa was also observed (IVW-OR: 1.001, 95% CI: 1.000-1.003, *P* = 0.045; weighted median OR: 1.002, 95% CI: 1.000-1.003, *P* = 0.043). MR scatter plots and forest plots for CRP and PGE-2 as they related to PCa risk are presented in **Figures 4**, **5**.

**Table 3:** Inflammatory cytokines significantly associated with PCa.

Cytokines	Phenotype	Number of SNPSs	Variance in Cytokines levels Explained,%	OR_IVW_(95%CI)	P Value	FDR
CRP		4	2.5	1.001(1.000-1.003)	0.043	0.6312369
PGE-2		3	2.7	1.001(1.000-1.003)	0.045	0.4977888

CI, confidence interval; FDR, false discovery rate; IVW, inverse variance; OR, odds ratio.

In total, 12 significant SNPs were identified as IVs for PCa. SCF (OR: 0.001, 95%CI: 0.00-0.44, *P* = 0.025) was selected as an outcome measure for inflammatory cytokines. No significant values were obtained from MR-Egger (*P* = 0.974422) or weighted median (*P* = 0.310249) analyses. No other inflammatory factors exhibited substantial results when used as outcome variables to assess the potential for reverse causality (**Supplementary Table 3**).

## 4 Discussion

In this investigation, a two-sample MR methodology was utilized to investigate the association between the risk of PCa and 44 distinct inflammatory biomarkers. While PCa was found to be significantly associated with the levels of CRP and PGE-2, no reverse causality was detected between PCa and any of the analyzed inflammatory factors. This thus suggests a potential causal role for PGE-2 and CRP in developing PCa. The absence of significant reverse causality aligns with our understanding of cancer biology, where chronic inflammation is thought to contribute to cancer initiation and progression.

The results of this study offer support for prior data demonstrating the significant role that inflammatory responses play in the pathogenesis of PCa. Notably, the abnormal expression of the inflammatory factors CRP and PGE-2 has previously been linked to a greater risk of PCa and a less favorable patient prognosis. The results of the present MR analysis further validate this relationship, offering additional evidence in favor of a causal link between inflammation and PCa.

Numerous investigations have concentrated on the correlation of inflammatory factors with the prevalence of PCa. The platelet-to-lymphocyte count ratio (PLR) is a highly accurate and clinically relevant predictor of PCa, and it can also predict Gleason scores such that patients exhibiting higher PLR levels face a greater risk of mortality. Levels of PCT are also reportedly higher in PCa patients, so it may offer diagnostic utility. Increased CRP levels have also been proposed as an independent predictor of worse overall survival (OS) in individuals with metastatic PCa (10). The CRP to albumin ratio (CAR) and time to castration resistance (TTCR) are both independent prognostic indices associated with castration-resistant PCa, and combining CAR and TTCR can allow for a more accurate predictive assessment of metastatic castration-resistant PCa patients (24). CRP levels have also been confirmed to be related to poorer PCa patient OS in a meta-analysis (25). Patients facing higher rates of tumor-associated mortality also tend to exhibit higher levels of specific cytokines (e.g., IL-6, IL-17A, IL-22, and transforming growth factor-β (TGF-β)) (26).

Several studies have shown that inflammatory cytokines, including IL-1, IL-6, TNF-α, and IL-25, are crucial in increasing tumor-associated inflammation, metastasis, angiogenesis, and apoptotic induction (27–29). Despite the wealth of observational evidence supporting a potential link between these cytokines and PCa, establishing a causal relationship remains difficult owing to limitations associated with classical epidemiological techniques. Observational studies cannot account for reverse causality or confounding factors, so it is impossible to clarify whether detected relationships between exposures and diseases of interest are truly causal. The higher levels of many inflammatory cytokines in PCa patients may thus be indicative of the etiology of this disease, the infection status of patients, treatment-related side effects, or other concurrent pathological immune reactions that traditional observational studies cannot differentiate among (30).

The results of the present MR study suggest that CRP and PGE-2 may significantly contribute to PCa development. PGE-2 production is mediated by the cyclooxygenase (COX-1/2) and microsomal prostaglandin E synthase-1 (mPGES-1) enzymes, and it serves as a pro-inflammatory mediator involved in cardiovascular disease, rheumatoid arthritis, and various cancers. Prostaglandins are derived from the cell membrane component arachidonic acid. As intracellular calcium levels rise, this activates cytosolic phospholipase A2 (PLA2) and triggers phospholipid degradation, generating free lysophospholipids and arachidonic acid. COX-1/2 then converts arachidonic acid into PGH2, after which it can undergo further conversion into a range of prostaglandins and thromboxanes that include PGE-2, PGD2, PGI2, PGF2α, and TXA2 (31). The mPGES-1 enzyme is responsible for the production of PGE-2 from PGH2, with the activity of mPHES-2S-1 being inducible and dependent on the presence of glutathione (GSH) for activation (32).

Prostaglandins signal through particular receptors to influence intracellular cAMP and Ca^2+^ levels, thereby regulating smooth muscle contraction in blood vessels. There is some evidence suggesting that PGE-2 signaling and chronic human papillomavirus infection can compromise immune function and contribute to cervical cancer development (33). PGE-2 signaling enhances the intrinsic tumorigenic ability to increase breast tumor stem cells (34). CRP is an evolutionarily conserved plasma protein encoded on chromosome 1 by a single intron with a signal peptide and a mature protein-coding region. In hepatic cells, CRP transcription is primarily regulated by IL-6, with IL-1 potentially augmenting these regulatory effects (35). Like many other inflammatory mediators, CRP exhibits pleiotropic pro- and anti-inflammatory characteristics, including the ability to stimulate IL-1 receptor antagonist expression (36), enhance anti-inflammatory IL-10 release, and suppress IFN-γ production (37).

Concerning its pro-inflammatory activity, higher serum levels of CRP have been linked to the overall and progression-free survival of patients with ovarian cancer, with a close relationship between the levels of CRP, tumor aggressiveness, and disease progression in these patients (38). CRP levels have also been tied to the odds of PCa development in patients exhibiting high levels of PSA, while also related to a greater chance of biochemical recurrence after undergoing curative treatment for localized PCa and to worse OS in patients with advanced disease (25, 39). Many studies have confirmed that both PGE-2 and CRP are important in the context of PCa (40, 41), in line with the present findings. Although the effect sizes observed in our study are modest, the chronic inflammatory process may play a pivotal role in carcinogenesis. Long-term exposure to elevated levels of inflammatory markers such as CRP and PGE-2 might create a cellular environment that favors tumor initiation and progression. Previous studies have suggested that chronic inflammation can lead to DNA damage, promote angiogenesis, and impair immune surveillance, all of which could contribute to the development of PCa (42, 43). While debate remains regarding the precise relationships between inflammatory cytokines and the onset and progression of PCa, the reasons for unsatisfactory treatment outcomes remain uncertain. The present results support a role for both PGE-2 and CRP in the incidence of PCa. Nevertheless, it is crucial to acknowledge that the impact on PCa may not be immediate, but rather facilitated by intermediary interactions (44, 45). Treatments targeting these factors may thus fail to abrogate disease progression. The use of immunomodulatory treatment strategies may be more efficacious if specific exposure-outcome associations with disease onset are observed.

The evidence indicating the involvement of PGE-2 and CRP in the pathogenesis of PCa underscores the necessity of integrating these biomarkers into risk assessment models. By identifying individuals with elevated levels of these markers, screening programs could be optimized to detect PCa at an earlier stage, thereby potentially improving treatment outcomes. Furthermore, several studies have demonstrated that PCa patients exhibiting elevated CRP levels tend to have poorer OS, cancer-specific survival (CSS), and progression-free survival (PFS) (25). Furthermore, the intermediary role of inflammation in disease progression indicates that conventional treatments may not sufficiently address the underlying inflammatory mechanisms. Consequently, targeting prostate cancers characterized by elevated CRP levels or increased PGE-2 production mediated by COX-1/2 with anti-inflammatory or immunomodulatory strategies could serve as a beneficial adjunct. For example, COX-2 inhibitors or other agents that specifically target inflammatory pathways may mitigate the chronic inflammatory state and potentially decrease the risk of PCa (46). Additionally, the application of immunomodulatory therapies warrants consideration.

Overall, this is the first MR study probing the causal link between PCa and the levels of 44 different inflammatory factors. However, these results are subject to certain limitations. For one, it was impossible to adequately test the second and third hypotheses owing to constraints associated with this MR analysis, potentially contributing to some bias. Sample sizes are different between cytokines which influence the power of genetic associations and the robustness of the MR analysis. In addition, while this study was based on two large-scale GWAS studies, subgroup analyses could not be performed owing to the absence of specific demographic information and supporting clinical records from analyzed subjects. Recognizing these constraints, future efforts will focus on incorporating broader demographic data and expanding genetic analyses to include a more diverse array of populations. This expansion is crucial to enhance the generalizability and applicability of our findings across different ethnicities. Thus, Caution is warranted when attempting to generalize these results to populations not of European ancestry. Additional research will be necessary to validate these findings and to clarify their applicability to diagnostic and treatment efforts. Finally, no significance was found in reverse causality analyses using inflammatory cytokines as outcome variables related to PCa, which could influence the credibility of the reverse causation results.

## 5 Conclusions

While the existing results highlighted an association of the levels of CRP and PGE-2 with the incidence risk of PCa, it is imperative to emphasize the necessity for additional research to substantiate and validate this intriguing finding. In particular, it will be essential to explore how CRP and PGE-2 influence PCa development and progression and clarify how they interact with other biomarkers. The potential impacts of different variables, including age, family history, and lifestyle, on these relationships should also be probed in greater detail. Further studies will also be necessary to clarify the potential applicability of these findings to the formulation of diagnostic and treatment strategies that can be deployed in clinical settings.

## Data Availability

The original contributions presented in the study are included in the article/**Supplementary Material**. Further inquiries can be directed to the corresponding authors.
